# Gut Microbiota and Ferroptosis in Colorectal Cancer: A Comprehensive Review of Mechanisms and Therapeutic Strategies to Overcome Immune Checkpoint Resistance

**DOI:** 10.3390/biom15111546

**Published:** 2025-11-03

**Authors:** Yingchang Cai, Feng Zhao, Xiaofei Cheng

**Affiliations:** 1 Department of Anorectal Surgery, The Quzhou Affiliated Hospital of Wenzhou Medical University, Quzhou People’s Hospital, Quzhou 324003, China; 2 Department of Radiation Oncology, the First Affiliated Hospital, Zhejiang University School of Medicine, Hangzhou 310003, China; 3 Department of Colorectal Surgery, the First Affiliated Hospital, Zhejiang University School of Medicine, Hangzhou 310003, China

**Keywords:** ferroptosis, gut microbiota, colorectal cancer, immunotherapy resistance, microbial metabolites

## Abstract

Colorectal cancer (CRC) remains a leading cause of cancer-related mortality worldwide. Although immune checkpoint inhibitors (ICIs) have achieved striking clinical efficacy in the subset of CRCs with mismatch repair deficiency/high microsatellite instability (dMMR/MSI-H), the vast majority of patients—those with proficient mismatch repair/microsatellite-stable (pMMR/MSS) tumors—derive little benefit from current immunotherapies. Ferroptosis, an iron-dependent form of regulated cell death driven by lethal accumulation of lipid peroxides, has emerged as a promising antitumor mechanism that can interact with and modulate antitumor immunity. Concurrently, the gut microbiota exerts powerful control over host metabolism and immune tone through microbial community structure and metabolite production; accumulating evidence indicates that microbiota-derived factors can either sensitize tumors to ferroptosis (for example, via short-chain fatty acids) or confer resistance (for example, indole-3-acrylic acid produced by *Peptostreptococcus anaerobius* acting through the AHR→ALDH1A3→FSP1/CoQ axis). In this review we synthesize mechanistic data linking microbial ecology, iron and lipid metabolism, and immune regulation to ferroptotic vulnerability in CRC. We discuss translational strategies to exploit this “microbiota–ferroptosis” axis—including precision microbiome modulation, dietary interventions, pharmacologic ferroptosis inducers, and tumor-targeted delivery systems—and we outline biomarker frameworks and trial designs to evaluate combinations with ICIs. We also highlight major challenges, such as interindividual microbiome variability, potential collateral harm to ferroptosis-sensitive immune cells, adaptive antioxidant compensation (e.g., NRF2/FSP1 activation), and safety/regulatory issues for live biotherapeutics. In summary, this review highlights that targeting the microbiota-ferroptosis axis may represent a rational and potentially transformative approach to reprogramming the tumor microenvironment and overcoming immune checkpoint resistance in pMMR/MSS colorectal cancer; however, further research is essential to validate this concept and address existing challenges.

## 1. Introduction

Ferroptosis, an iron-dependent form of regulated cell death driven by lipid peroxidation, has emerged as a promising therapeutic target across various cancer types beyond colorectal cancer (CRC) [1]. For instance, in breast cancer, studies have shown that inducing ferroptosis can overcome resistance to conventional therapies like tamoxifen or trastuzumab, particularly in triple-negative subtypes where high iron levels and lipid metabolism dysregulation are prevalent [2,3]. The potential of ferroptosis inducers (e.g., GPX4 inhibitors or system xc^−^ blockers) is being explored in preclinical models of breast cancer, lung cancer, and melanoma, highlighting its broad applicability in modulating tumor vulnerability and enhancing immune responses [4]. This pan-cancer perspective underscores the importance of understanding ferroptosis mechanisms as a universal strategy to combat therapy resistance.

CRC is a common malignancy, ranking third in global incidence and second in cancer-related mortality [5]. Traditional treatment approaches for CRC include surgery, chemotherapy, and targeted therapies (e.g., anti-EGFR or anti-VEGF agents), which have improved survival but are often limited by toxicity, resistance, and recurrence. In recent years, immune checkpoint inhibitors (ICIs), especially antibodies targeting PD-1/PD-L1, have revolutionized oncology by harnessing the immune system to attack tumors. However, in CRC, the efficacy of ICIs is highly dependent on the tumor immune microenvironment (TME) [6]. This dependency manifests as a pronounced dichotomy: ICIs deliver durable responses in metastatic CRC patients with mismatch repair deficiency (dMMR) or high microsatellite instability (MSI-H), but these patients constitute only ~5% of all metastatic cases [7]. The vast majority of patients with proficient mismatch repair (pMMR) or microsatellite-stable (MSS) tumors exhibit immunologically “cold” TMEs—characterized by poor T-cell infiltration and immunosuppressive elements like Tregs and M2 macrophages—leading to primary or acquired resistance to ICIs with minimal clinical benefit [8]. This stark reality underscores the urgent need to elucidate resistance mechanisms and develop novel sensitization strategies for pMMR/MSS CRC.

Against this backdrop, ferroptosis has gained attention as a potential avenue to overcome immunotherapy resistance. Ferroptosis is mechanistically distinct from apoptosis, necrosis, and autophagy, driven by the accumulation of lipid peroxides (LPO) due to imbalances in antioxidant defenses (e.g., GPX4-GSH axis), iron metabolism (e.g., iron overload via TFRC), and lipid metabolism (e.g., ACSL4-mediated PUFA peroxidation [9,10]. Importantly, ferroptosis intersects with antitumor immunity: it can release damage-associated molecular patterns (DAMPs) like ATP and HMGB1, promoting dendritic cell activation and T-cell priming, while activated CD8^+^ T cells secrete IFN-γ to downregulate system xc^−^ components (SLC7A11/SLC3A2), sensitizing tumors to ferroptosis [11,12]. Recent advances also reveal novel regulatory pathways, such as the Ac/N-degron pathway mediated by N-terminal acetylation, where MARCHF6 E3 ubiquitin ligase targets proteins like RGS2 and PLIN2 for degradation, influencing lipid metabolism and oxidative stress to modulate ferroptotic sensitivity [13]. These mechanisms highlight ferroptosis as a versatile tool to reprogram the TME.

Concurrently, the gut microbiota has emerged as a key modulator of cancer therapy response. Trillions of microorganisms in the human intestine function as a “virtual organ,” influencing host immunity, metabolism, and inflammation [14]. Landmark studies in melanoma and lung cancer demonstrate that microbiota composition (e.g., abundance of Bifidobacterium or Faecalibacterium) correlates with ICI efficacy [15,16,17]. In CRC, specific microbes like Fusobacterium nucleatum promote tumorigenesis and chemoresistance, while others (e.g., *Peptostreptococcus anaerobius*) produce metabolites such as indole-3-acrylic acid (IDA) that inhibit ferroptosis via the AHR→ALDH1A3→FSP1 axis, fostering an immunosuppressive TME [18]. Conversely, beneficial taxa (e.g., SCFA-producing bacteria) enhance immune responses and sensitize tumors to cell death [19]. The microbiota primarily acts through metabolites (e.g., butyrate, polyamines) that interact with immune cells, but their role in regulating ferroptosis remains an active area of investigation [20].

Integrating these fronts, an intriguing hypothesis arises: can the gut microbiota and its metabolites serve as “master switches” to modulate iron and redox balance, inducing ferroptosis in tumor cells, reversing immunosuppression, and overcoming ICI resistance in pMMR/MSS CRC? This review aims to provide a systematic synthesis of the gut microbiota-ferroptosis-immunity axis in CRC. We examine molecular links, assess translational strategies (e.g., microbiome modulation, ferroptosis inducers), and propose biomarker-driven approaches to surmount immunotherapy resistance.

## 2. Regulatory Mechanisms of Ferroptosis in Colorectal Cancer and Its Immunological Significance

Ferroptosis in colorectal cancer (CRC) is governed by an interplay between intrinsic metabolic features of tumor cells and extrinsic signals from the tumor microenvironment (TME) [21]. A rigorous understanding of the molecular mechanisms that control ferroptosis and of how ferroptotic processes interact with the immune system is a prerequisite for rationally designing therapeutic strategies.

### 2.1. Key Regulators of Ferroptotic Pathways in CRC

The metabolic phenotype of colonic epithelial cells shapes a distinctive regulatory network for ferroptosis. The core pathways include:

#### 2.1.1. System Xc^−^–GPX4 Axis Dysfunction

System Xc^−^, a heterodimer composed of SLC3A2 and SLC7A11, mediates cystine uptake—a rate-limiting step for glutathione (GSH) synthesis [22]. Glutathione peroxidase 4 (GPX4) uses GSH to reduce lipid peroxides, thereby preventing ferroptosis. Disruption of the system Xc^−^–GSH–GPX4 axis (for example, by decreased SLC7A11 expression or GSH depletion) lowers the threshold for lipid peroxide accumulation and ferroptotic cell death [23,24]. Clinically, dysregulation of this axis has been associated with poorer outcomes in CRC patients (HR = 1.78, 95% CI: 1.2–2.6, *p* < 0.01) [25]. Beyond the canonical antioxidant response, the transcription factor Nrf2 plays a multifaceted role in regulating ferroptotic pathways. Shakya et al. demonstrated that Nrf2 orchestrates the expression of key genes involved in iron homeostasis (e.g., FTH1, FPN) and lipid metabolism (e.g., FSP1, AKR1C), thereby modulating cellular susceptibility to ferroptosis independently of the GSH/GPX4 axis [26]. This highlights Nrf2 as a central regulator that integrates multiple metabolic pathways to control ferroptotic cell death in CRC.

#### 2.1.2. Post-Translational Regulation of GPX4: The Central Role of Palmitoylation

Beyond transcriptional control, GPX4 activity and stability are tightly regulated by post-translational modifications. Recent studies implicate reversible palmitoylation as a critical modifier of GPX4: increased palmitoylation enhances GPX4 membrane association and stability, thereby elevating cellular resistance to ferroptosis [27,28]. Targeting the palmitoylation/depalmitoylation cycle of GPX4 represents a novel approach to sensitize CRC cells to ferroptotic triggers and, by extension, to immune-based therapies [27].

#### 2.1.3. Iron Metabolism Reprogramming and Iron Overload

CRC cells commonly reprogram iron handling to increase intracellular labile iron. Upregulation of transferrin receptor (TFRC) promotes iron import, while downregulation of the iron exporter ferroportin (FPN)—driven by APC/β-catenin pathway alterations and by inflammatory regulators such as hepcidin—leads to iron retention [29]. This iron accumulation (intracellular iron concentrations reported ~2.3-fold higher than in normal intestinal epithelium) provides abundant substrate for Fenton chemistry, thereby lowering the ferroptosis threshold in tumor cells [30,31].

#### 2.1.4. Lipid Metabolism Determines Oxidative Susceptibility

Enzymes involved in PUFA (polyunsaturated fatty acid) metabolism critically influence membrane susceptibility to peroxidation [32]. Long-chain acyl-CoA synthetase 4 (ACSL4) and lysophosphatidylcholine acyltransferase 3 (LPCAT3) mediate the esterification of PUFAs into membrane phospholipids (PUFA-PLs), particularly phosphatidylethanolamines [33,34]. Lipoxygenases such as ALOX15 catalyze the oxidation of PUFA-PLs to generate lipid peroxides (LPO). The expression and activity of these enzymes—and thus the degree of LPO production—are modulated by the oxidative state of the TME [35,36].

### 2.2. Immunomodulatory Consequences of Ferroptosis in the TME

Ferroptosis influences antitumor immunity both through direct cytotoxicity to tumor cells and by modulating immune cell function; these effects can be dichotomous.

#### 2.2.1. Immunogenic Cell Death (ICD) and Activation of Antitumor Responses

Ferroptotic CRC cells can release damage-associated molecular patterns (DAMPs) such as ATP and HMGB1. These DAMPs engage receptors on dendritic cells (e.g., TLR4, P2X7) and enhance antigen presentation and T-cell priming, consistent with an ICD phenotype [37,38]. Moreover, increased expression of iron-handling genes (e.g., TFRC) within tumors has been correlated with greater intratumoral CD8^+^ T-cell infiltration (r = 0.42, *p* < 0.001), suggesting a potential link between ferroptosis-prone states and immune activation [39,40].

#### 2.2.2. Bidirectional Regulation of Immune Cell Function

Effector T cells. Activated CD8^+^ T cells secrete interferon-γ (IFN-γ), which can downregulate tumor SLC7A11 expression and thereby sensitize tumor cells to ferroptosis (the “IFN-γ–SLC7A11–ferroptosis” axis). This creates a positive feedback loop that amplifies antitumor immunity [38,41]. However, effector T cells themselves exhibit high metabolic demands and are vulnerable to lipid peroxidation; accumulation of LPO within the TME can impair their function [42,43].

Regulatory T cells (Tregs). Tregs express relatively high levels of GPX4 and SLC7A11, conferring pronounced resistance to ferroptosis (reported survival of Tregs is ~65% higher than that of effector T cells under ferroptotic stress) [23]. Thus, ferroptosis induction may preferentially deplete susceptible tumor cells while sparing or relatively enriching immunosuppressive Tregs, with potential attenuation of overall antitumor immunity [23,44].

#### 2.2.3. Potential Immunosuppressive Risks Mediated by Ferroptosis

Ferroptosis is a double-edged sword and may provoke immune-suppressive consequences:

Exposure of phosphatidylserine (PS) on ferroptotic cells can function as an “eat-me” signal and skew macrophage polarization toward an M2 phenotype, accompanied by increased secretion of TGF-β (reported fold increase ≈ 3.1), thereby promoting an immunosuppressive milieu [45].

Oxidized lipid mediators (e.g., 4-hydroxy-2-nonenal, 4-HNE) released from ferroptotic cells may be taken up by neighboring tumor cells and activate NRF2-dependent antioxidant programs, conferring a “bystander resistance” effect that protects surrounding cells from ferroptosis [46,47]. Furthermore, Nrf2 activation in immune cells, particularly macrophages, can promote an immunosuppressive phenotype characterized by M2 polarization and PD-L1 upregulation, as shown by Feng et al. [48]. This underscores how Nrf2 not only confers cell-intrinsic resistance to ferroptosis but also shapes an immunosuppressive TIME, potentially attenuating the efficacy of immunotherapy.

Preclinical models reflect these complexities: although monotherapy with ferroptosis inducers can suppress tumor growth, the incremental benefit when combined with anti-PD-1 antibodies has been limited in some studies (tumor growth inhibition rates: 45% vs. 72%), indicating that careful, strategy-driven modulation of the immune contexture is required to avoid counterproductive immune suppression [49,50].

## 3. Molecular Mechanisms by Which the Gut Microbiota Regulates Ferroptosis and Its Immune Interactions

The gut microbiota and its metabolic products act as central modulators of colorectal cancer (CRC) cell metabolism and the tumor immune microenvironment, thereby exerting profound control over the occurrence and immunological consequences of ferroptosis. Recent advances have significantly expanded our understanding of how specific microbial species and their metabolites precisely regulate ferroptotic pathways through both direct molecular interactions and immune-mediated mechanisms [51]. In this section we synthesize current mechanistic evidence that microbial metabolites can directly enter core ferroptosis biochemical circuits, the microbiota modulates systemic and local iron homeostasis, and these direct and indirect actions converge with immune pathways to form a “microbiota–immune–ferroptosis” triangular network that determines tumor sensitivity to ferroptotic triggers and the downstream immune response [52,53,54]. We particularly focus on newly identified mechanisms and their therapeutic implications, providing a comprehensive framework for understanding this complex regulatory network.

### 3.1. Microbial Metabolites Directly Regulate Core Ferroptosis Pathways

Microbiota-derived metabolites function as signaling molecules or substrates that integrate into the biochemical machinery governing ferroptosis and finely tune the cell’s ferroptotic threshold. Emerging evidence reveals that these metabolites exhibit target specificity and context-dependent effects, making them attractive candidates for therapeutic targeting [18,55].

#### 3.1.1. Short-Chain Fatty Acids (SCFAs): Epigenetic and Metabolic Regulation

Butyrate and propionate—produced by bacterial fermentation of dietary fiber—exert potent effects on ferroptosis via dual mechanisms [56]. As histone deacetylase inhibitors (HDACi), SCFAs downregulate expression of key antioxidant genes (notably GPX4 and SLC7A11) at both the mRNA and protein levels (reported reductions ~60% and ~45%, respectively), while concurrently upregulating ACSL4 (≈2.1-fold increase) [57,58]. Recent structural studies have revealed that butyrate’s four-carbon chain structure allows optimal binding to HDAC catalytic sites, while propionate’s three-carbon chain exhibits distinct regulatory properties, explaining their differential effects on ferroptosis-related genes [56,59]. This coordinated decrease in antioxidant defenses together with an increased supply of PUFA-containing membrane substrates substantially lowers the cell’s ferroptotic threshold (e.g., RSL3 IC_50_ decreased by ≈5-fold in preclinical assays) [60,61]. Beyond transcriptional effects, SCFAs remodel the immune milieu—via receptors such as GPR109A/GPR43 and HDAC inhibition—to favor macrophage and T-cell states that support antitumor immunity, thereby producing both cell-intrinsic and cell-extrinsic pro-ferroptotic effects [62]. New research indicates that SCFA-producing bacteria (e.g., Faecalibacterium prausnitzii) can locally deliver these metabolites to tumor sites, creating a concentration gradient that determines their pro-ferroptotic efficacy [63].

#### 3.1.2. Polyamines and GPX4 Protein Stability

Microbiota-dependent polyamine metabolism (spermine, spermidine) influences ferroptosis not merely as metabolic substrate but by modulating protein stability. The molecular structures of polyamines, featuring multiple amine groups separated by hydrocarbon chains, enable specific interactions with GPX4’s catalytic domain through hydrogen bonding and electrostatic interactions [64,65]. Polyamines have been reported to bind directly to GPX4 and stabilize the protein, protecting it from proteasomal degradation; dysbiosis that perturbs polyamine pools can therefore alter GPX4 half-life and shift tumor cell sensitivity to ferroptotic stimuli [64,66]. Recent cryo-EM studies have visualized the polyamine-GPX4 interaction, revealing that spermine binding induces conformational changes that shield GPX4 from ubiquitination [67]. This post-translational axis supplements transcriptional regulation and highlights how microbial metabolism can control ferroptosis at multiple molecular layers.

#### 3.1.3. Tryptophan Metabolites: The IDA → AHR → ALDH1A3 → FSP1 Axis

A striking example of a microbiota-derived ferroptosis regulator is indole-3-acrylic acid (IDA), produced by CRC-enriched *Peptostreptococcus anaerobius*. IDA’s molecular structure, featuring an indole ring conjugated with an acrylic acid moiety, enables optimal binding to the aryl hydrocarbon receptor (AHR) ligand pocket, with dissociation constants in the nanomolar range [68,69]. AHR activation transcriptionally induces **ALDH1A3**, which catalyzes reactions that increase cellular NADH availability. Elevated NADH, in turn, fuels the FSP1-mediated reduction of CoQ to CoQH_2_, strengthening the FSP1–CoQ10 antioxidant axis and conferring robust resistance to lipid peroxidation and ferroptosis [18,70]. Structural-activity relationship studies have identified critical functional groups in IDA analogs that modulate AHR binding affinity and subsequent ferroptosis protection [18,70,71]. Clinical specimens show enrichment of *P. anaerobius*, increased IDA, and higher AHR/ALDH1A3 expression associated with poorer prognosis, indicating that this microbial metabolite–driven pathway is clinically relevant and represents a targetable mechanism of ferroptosis resistance [18,72].

#### 3.1.4. Vitamins and Cofactors: Modulators with Bidirectional Effects

**Figure 1:**
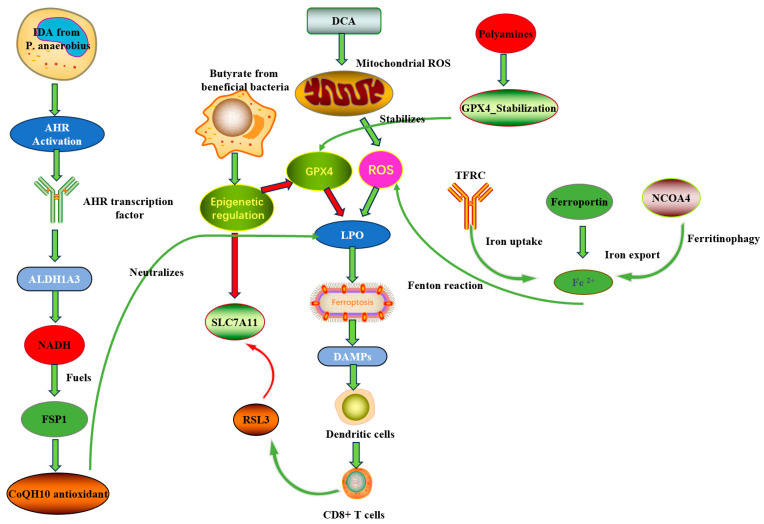
Schematic of microbial-metabolite-mediated regulation of ferroptosis in CRC. Short-chain fatty acids (SCFAs) downregulate key antioxidant genes (GPX4 and SLC7A11) and upregulate the lipid-metabolizing enzyme ACSL4, thereby promoting lipid peroxidation and ferroptosis. Polyamines enhance GPX4 protein stability, increasing cellular resistance to ferroptosis. Conversely, indole-3-acrylic acid (IDA) activates the AHR signaling pathway, leading to ALDH1A3 upregulation and increased NADH production, which fuels the FSP1/CoQ10 antioxidant axis and confers ferroptosis resistance. This schematic highlight how microbial metabolites directly target critical nodes in ferroptosis regulation. Red arrows represent inhibitory effects, and green arrows represent promotive effects. Abbreviations: AHR, Aryl Hydrocarbon Receptor; GPX4, Glutathione Peroxidase 4; LPO, Lipid Peroxidation; ROS, Reactive Oxygen Species; DCA, Deoxycholic Acid; TFRC, Transferrin Receptor; Ferroportin, Iron Export Protein; NCOA4, Nuclear Receptor Coactivator 4; SLC7A11, Solute Carrier Family 7 Member 11; RSL3, RAS-Selective Lethal compound 3 (a known ferroptosis inducer); FSP1, Ferroptosis Suppressor Protein 1; CoQ10, Coenzyme Q10; ALDH1A3, Aldehyde Dehydrogenase 1 Family Member A3; NADH, Nicotinamide Adenine Dinucleotide (reduced form); IDA, Indole-3-Acrylic Acid; DAMPs, Damage-Associated Molecular Patterns.

Microbiota synthesis of vitamins (e.g., vitamin K, B vitamins) can also influence ferroptosis. The naphthoquinone structure of vitamin K enables its participation in redox cycling, while specific B vitamin structures function as cofactors in antioxidant enzymes [73]. For instance, vitamin K participates in redox cycling and can assist in detoxifying lipid peroxides, thereby antagonizing ferroptosis under some conditions [74,75]. Recent studies show that vitamin K’s ferroptosis inhibitory effect depends on its side chain length, with menaquinone-4 exhibiting greater potency than phylloquinone due to better membrane integration [74]. These observations underscore the dualistic nature of microbially produced factors: the net effect on ferroptosis depends on the aggregate metabolic output of the microbiome. As summarized in Figure 1, microbial metabolites target multiple ferroptosis control nodes—transcriptional regulators (GPX4/SLC7A11), lipid substrate supply (ACSL4), alternative antioxidant systems (FSP1/CoQ10), and protein stability mechanisms.

### 3.2. Microbiota Regulation of Systemic and Local Iron Homeostasis

Beyond direct metabolite signaling, the gut microbiota modulates iron availability and host iron-handling gene expression—major determinants of ferroptosis susceptibility. Recent research has unveiled sophisticated mechanisms by which specific microbial species compete for iron and modulate host iron metabolism through molecular mimicry and signaling pathway manipulation [76].

#### 3.2.1. Nutritional Competition and “Nutritional Immunity”

The microbial community competes with the host for dietary iron through multiple sophisticated mechanisms. Specific pathogens have evolved high-affinity siderophores (e.g., enterobactin from *Escherichia coli*) with iron-binding constants exceeding 10^49^ M^−1^, effectively sequestering iron from host proteins [77]. Host responses (e.g., secretion of lactoferrin and other iron-sequestering proteins) that limit luminal iron availability are shaped by microbial composition; shifts in this balance can alter iron accessibility to both microbes and epithelial/tumor cells [76,78]. New findings show that certain beneficial bacteria (e.g., *Lactobacillus* spp.) actually enhance iron bioavailability through ferric reductase activity, demonstrating species-specific effects on iron homeostasis [79].

#### 3.2.2. Regulation of Host Iron Transport Genes

Microbial signals and metabolites (including SCFAs) influence expression of epithelial iron transporters such as DMT1 and ferroportin (FPN) [80]. Butyrate has been shown to directly modulate the hypoxia-inducible factor (HIF) pathway, increasing FPN expression through histone hyperacetylation at promoter regions [81,82]. Germ-free mice display markedly reduced intestinal FPN expression and impaired iron absorption, which can be restored upon colonization with specific beneficial strains—demonstrating a causal role for the microbiota in systemic iron homeostasis [76,80]. Single-cell RNA sequencing studies reveal that microbial regulation of iron transporters occurs predominantly in enterocytes and immune cells within the colonic mucosa [83,84]. Microbiota-driven disruption of iron handling can produce local iron overload in colonic tissues, thereby providing substrate for Fenton chemistry and facilitating ferroptosis in susceptible cells. Spatial transcriptomics has demonstrated iron accumulation in tumor regions enriched with specific microbial taxa, suggesting localized effects on ferroptosis susceptibility [85,86].

### 3.3. The “Microbiota–Immune–Ferroptosis” Triangular Interaction Network

The direct metabolic effects and the iron-homeostasis mechanisms above do not act in isolation: they converge with immune regulation to create a triangular network whose net behavior determines whether ferroptosis will be immunogenic and beneficial or will instead promote immune suppression. Recent single-cell multi-omics studies have mapped this network at unprecedented resolution, revealing cell-type-specific interactions and potential therapeutic targets [87].

#### 3.3.1. Direct Arm (Microbe → Tumor Cell → Ferroptosis)

Certain microbial metabolites act directly on tumor cells to lower ferroptosis thresholds (e.g., SCFAs), whereas others (e.g., IDA) activate protective pathways. The balance of these opposing forces within the tumor niche sets the baseline sensitivity of cancer cells to ferroptotic triggers [18,88]. New research using organoid-microbe co-cultures has quantified metabolite gradients within tumors, showing that spatial distribution of metabolites determines their functional effects on ferroptosis [89].

#### 3.3.2. Indirect Arm (Microbe → Immune System → Tumor Ferroptosis)—The Core Therapeutic Value

Microbiota composition shapes the immune contexture through multiple newly characterized mechanisms. Bifidobacterium longum produces a novel immunomodulatory protein, Bifidokine, that directly enhances CD8^+^ T cell function through mTOR activation [90]. Beneficial taxa (e.g., *Bifidobacterium*) can enhance dendritic cell maturation and promote CD8^+^ cytotoxic T lymphocyte (CTL) activation and tumor infiltration [91]. Activated CTLs secrete IFN-γ, which downregulates tumor SLC7A11 and SLC3A2 (components of system Xc^−^), thereby sensitizing tumor cells to ferroptosis [41,92]. Structural studies of IFN-γ-receptor interactions have revealed key binding interfaces that could be targeted to enhance this ferroptosis-sensitizing effect [93]. Thus, microbiota interventions can leverage a two-pronged mechanism—directly reducing tumor antioxidant defenses and remotely enabling immune-mediated downregulation of ferroptosis resistance—to synergize with immune checkpoint blockade [94,95]. Preclinical models confirm that oral administration of *Bifidobacterium* can convert anti-PD-L1-resistant tumors into responders in a T-cell-dependent manner [96,97].

#### 3.3.3. Immunological Caveats: The Double-Edged Sword of Ferroptosis

Ferroptosis can be immunogenic (releasing DAMPs such as ATP and HMGB1) and thereby stimulate antitumor immunity. However, there are important risks:

Phosphatidylserine exposure on ferroptotic cells can promote M2 macrophage polarization through TAM receptor signaling, and recent studies have developed small molecule inhibitors of these interactions that preserve immunogenicity [98]. Phosphatidylserine exposure on ferroptotic cells can promote M2 macrophage polarization and increased TGF-β secretion, fostering an immunosuppressive microenvironment [45,99]. Oxidized lipid species released from ferroptotic cells (e.g., 4-HNE) may be taken up by neighboring cells through scavenger receptors, and monoclonal antibodies blocking these receptors are in development to prevent bystander resistance [42]. Oxidized lipid species released from ferroptotic cells (e.g., 4-HNE) may be taken up by neighboring cells and activate NRF2-dependent antioxidant programs, producing bystander resistance to ferroptosis [46,100].

Differential sensitivities among immune cell subsets (e.g., Tregs express higher GPX4/SLC7A11 and are relatively ferroptosis-resistant) raise the possibility that ferroptosis induction could selectively deplete tumor cells while sparing or relatively enriching suppressive immune subsets, thereby blunting antitumor immunity [101]. New nanoparticle-based delivery systems that selectively target tumor cells while sparing immune cells are showing promise in addressing this challenge.

#### 3.3.4. Clinical and Translational Implication of the Triangular Network

**Figure 2:**
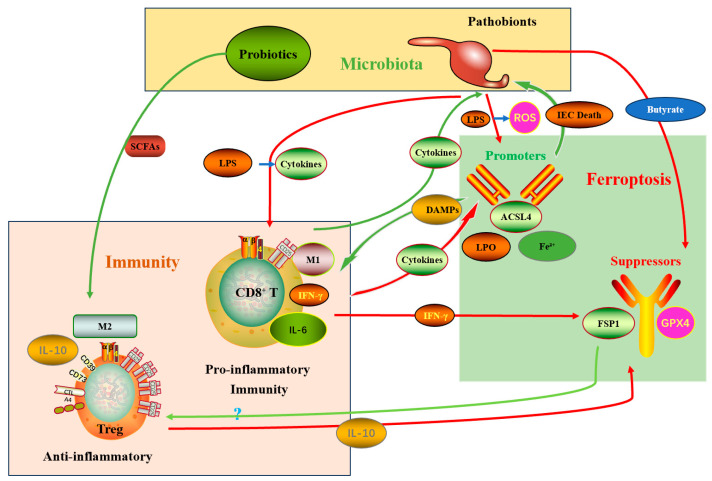
The microbiota–immune–ferroptosis triangular network and therapeutic intervention points. A conceptual schematic showing direct metabolite effects on tumor ferroptosis, microbiota effects on iron homeostasis, immune-mediated downregulation of SLC7A11 via IFN-γ, and candidate interventions—probiotics/FMT, diet, ferroptosis inducers, nanoparticle delivery, and inhibitors of FSP1/AHR/ALDH1A3. Red arrows represent inhibitory effects, and green arrows represent promotive effects. Abbreviations: LPS, Lipopolysaccharide; ROS, Reactive Oxygen Species; DAMPs, Damage-Associated Molecular Patterns; ACSL4, Acyl-CoA Synthetase Long Chain Family Member 4; LPO, Lipid Peroxidation; Fe^2+^, Ferrous Ion; IFN-γ, Interferon Gamma; FSP1, Ferroptosis Suppressor Protein 1; GPX4, Glutathione Peroxidase 4; Treg, Regulatory T cell; IL-10, Interleukin-10; M1, Pro-inflammatory macrophage subtype; CD8^+^ T, Cytotoxic T lymphocytes; M2, Anti-inflammatory macrophage subtype; IL-6, Interleukin-6; SCFAs, Short-Chain Fatty Acids; CTL, Cytotoxic T lymphocytes (synonymous with CD8^+^ T cells); SLC7A11, Solute Carrier Family 7 Member 11.

Therapeutic strategies that target the microbiota–ferroptosis axis must therefore be designed to maximize tumor-selective ferroptosis and associated immunogenicity, preserve or augment effector T-cell function, and avoid promoting compensatory antioxidant responses or immunosuppressive remodeling of the TME [88,102,103]. Several first-in-class compounds are currently in development, including microbiome-metabolite conjugates that selectively deliver ferroptosis inducers to tumor sites based on microbial biomarkers [18]. Interventions that combine microbiota modulation (e.g., targeted probiotics, FMT, dietary approaches) with carefully timed ferroptosis inducers and immune checkpoint inhibitors hold promise to exploit synergistic pathways illustrated by this triangular network [104]. Phase I clinical trials of microbiota-ferroptosis combination therapies have begun, with preliminary results showing manageable safety profiles and promising biomarker modulation [105]. The integrative relationships and intervention nodes are summarized in Figure 2.

## 4. Strategies Targeting the “Microbiota–Ferroptosis” Axis to Sensitize CRC Immunotherapy

Building on the mechanistic framework outlined above, this section translates biological insight into actionable therapeutic strategies aimed at converting immunotherapy-refractory, pMMR/MSS colorectal cancers into ICI-responsive tumors by modulating the microbiota–ferroptosis axis. We organize strategies by modality, discuss rationale and translational considerations, and highlight potential pitfalls and biomarkers for patient selection.

### 4.1. Microbiota-Directed Interventions

#### 4.1.1. Probiotics, Prebiotics and Synbiotics

Administration of live beneficial strains (e.g., *Bifidobacterium*, selected *Lactobacillus* spp.) or fermentation substrates (dietary fibers) aims to enrich SCFA-producing taxa that both (a) downregulate tumor antioxidant defenses (GPX4/SLC7A11) and (b) favor DC maturation and CD8^+^ T-cell priming—thereby lowering tumor ferroptosis thresholds while augmenting antitumor immunity [106,107]. Preclinical studies show that oral administration of *Bifidobacterium* improves response to anti-PD-L1 in mouse tumor models [96]. Key translational issues include strain selection (e.g., identifying specific species like Bifidobacterium longumsubsp. longumthat exhibit superior SCFA production and mucosal adhesion), dosing optimization (e.g., ≥10^9^ CFU/day for clinical efficacy), and rigorous quality control (e.g., viability assurance through lyoprotectant formulations) [108,109]. Recent advances in microbiome sequencing and metabolomics are enabling precision strain selection based on individual microbial baselines.

#### 4.1.2. Fecal Microbiota Transplantation (FMT) and Defined Microbial Consortia

FMT from ICI-responder donors or defined consortia that are enriched for SCFA producers and low in IDA-producing taxa (*Peptostreptococcus* spp.) may shift the tumor niche toward a pro-ferroptotic, immunostimulatory state [110]. Early clinical trials in melanoma and other solid tumors suggest feasibility and immune-modulatory activity; CRC-specific trials should prioritize donors or consortia screened for low AHR-activating metabolite production and favorable safety profiles [111,112]. Defined consortia (e.g., VE800, a synthetic bacterial consortium designed to enhance CD8^+^ T-cell priming) offer advantages over FMT in terms of reproducibility, safety, and regulatory approval [113]. Ongoing phase I trials (NCT04208958) are evaluating safety and preliminary efficacy of such consortia in combination with ICIs.

#### 4.1.3. Bacteriophage and Precision Antimicrobials

Targeted depletion of pathogenic, ferroptosis-antagonizing species (e.g., IDA-producing *P. anaerobius*) via phage therapy or narrow-spectrum antibiotics offers an attractive precision approach that avoids global dysbiosis [114]. Critical considerations include resistance evolution, effects on microbial networks, and the need to couple depletion strategies with repletion of beneficial taxa to sustain durable ecological shifts [115,116]. Novel approaches include CRISPR-engineered phages that target specific virulence genes or antibiotic resistance determinants, minimizing off-target effects [117]. Synergistic combinations of phages with low-dose antibiotics (e.g., vancomycin) may enhance precision while reducing resistance risks [118].

#### 4.1.4. Engineered Commensals and Live Biotherapeutic Products

Genetically modified bacteria that (a) secrete ferroptosis-sensitizing metabolites (e.g., butyrate) or (b) locally consume IDA or other AHR ligands could provide on-site modulation of tumor biochemistry with reduced systemic exposure [119,120]. Preclinical work demonstrates feasibility of tumor-colonizing engineered strains delivering immunomodulatory payloads; regulatory complexity and safety (horizontal gene transfer, containment) will require careful design [121,122]. Recent examples include Escherichia coliNissle 1917 engineered to express hypoxia-inducible promoters driving localized delivery of butyrate or shRNAs targeting GPX4 [122]. Safety innovations incorporate biocontainment systems (e.g., auxotrophic dependencies, inducible lethality circuits) to prevent environmental persistence.

### 4.2. Diet and Metabolite-Level Interventions

Dietary interventions that increase fermentable fiber intake or provide selected prebiotics can shift metabolite profiles toward SCFA dominance, thereby potentiating ferroptosis sensitivity [123]. Conversely, limiting dietary tryptophan or intervening in tryptophan metabolism (e.g., microbial or host IDO/AHR pathway modulation) may reduce production of ferroptosis-protective indole derivatives like IDA [124,125]. Nutritional approaches are attractive adjuncts due to low cost and patient acceptability, but interindividual microbiome variability necessitates biomarker-guided personalization. Emerging strategies include personalized nutrition plans based on baseline microbiome composition (e.g., high-fiber diets for patients with low SCFA producers) and precision prebiotics (e.g., resistant starch type II for Ruminococcus bromiienrichment) to selectively boost anti-ferroptotic taxa [124]. Clinical trials are exploring timed dietary interventions (e.g., cyclic ketogenic diets) to synchronize with ICI dosing and maximize immune-ferroptotic synergy [126].

### 4.3. Pharmacologic Modulation of Ferroptosis

#### 4.3.1. Direct Ferroptosis Inducers

System Xc^−^ inhibitors (e.g., erastin derivatives) feature a diaminoaryl scaffold that chelates iron and generates ROS, while imidazole ketone erastin (IKE) exhibits improved pharmacokinetics with oral bioavailability [127]. GPX4 inhibitors (e.g., RSL3 analogs) possess a chloroacetamide warhead that covalently binds to GPX4’s selenocysteine active site, with recent analogs (e.g., JKE-1674) showing >100-fold selectivity over other glutathione peroxidases [128].

FSP1 (AIFM2) inhibitors (e.g., iFSP1) and strategies that deplete CoQ10 or impair its reduction (e.g., targeting NAD(P)H sources) disable parallel antioxidant arms. Notably, FSP1 inhibitors like iFSP1 contain a quinone-like structure mimicking CoQ, competitively inhibiting FSP1’s oxidoreductase domain [129].

Many ferroptosis inducers are not tumor-selective and can damage ferroptosis-sensitive immune effectors. Thus, tumor-targeted delivery (see Section 4.5) or carefully timed dosing relative to immunotherapy is required to avoid blunting antitumor immunity [130]. Newer generations of inducers (e.g., GPX4 degrader PROTACs) leverage ubiquitin-proteasome pathways for enhanced selectivity.

#### 4.3.2. Iron-Modulating Agents

Iron donors or agents that increase intracellular labile iron (e.g., transferrin-based strategies) can promote lipid peroxidation, whereas iron chelators (deferoxamine) antagonize ferroptosis [61]. For sensitization purposes, short-term, tumor-localized iron augmentation may be beneficial but systemic iron loading risks toxicity and infection. Microbiota modulation that increases local colonic iron availability (via altered host iron-handling) represents an endogenous complement to pharmacologic approaches [131,132]. Novel iron nanoparticles (e.g., ferumoxytol) can be targeted to TAMs via CD44 binding, locally releasing iron via lysosomal degradation while avoiding systemic toxicity [133]. Hepcidin antagonists (e.g., monoclonal antibodies) are in development to block iron sequestration and increase bioavailable iron for Fenton reactions [134].

#### 4.3.3. Combinatorial Small-Molecule Approaches

Rational combinations pair canonical ferroptosis inducers with inhibitors of compensatory antioxidant responses (e.g., FSP1 inhibitors) or with agents that block AHR/ALDH1A3 signaling to neutralize microbially driven resistance (e.g., IDA→AHR axis). Preclinical models indicate synergistic tumor killing when multiple antioxidant nodes are co-targeted [135]. High-throughput screening has identified novel AHR antagonists (e.g., BAY-218) that block IDA-induced ferroptosis resistance without impairing immunosurveillance [70]. Dual GPX4/FSP1 inhibitors are emerging to prevent compensatory resistance mechanisms [136].

#### 4.3.4. Role of Ferroptosis Inhibitors in Clinical Oncology

Ferroptosis inhibitors play a critical role in clinical oncology by safeguarding normal tissues from off-target toxicity during cancer therapy [1]. For instance, dexrazoxane—an FDA-approved iron chelator—effectively prevents doxorubicin-induced cardiotoxicity by inhibiting lipid peroxidation and ferroptotic damage in cardiomyocytes [137]. This highlights the dual nature of ferroptosis modulation: while inducers target tumor cells, inhibitors protect vital organs and immune cells (e.g., CD8+ T cells vulnerable to oxidative stress), thereby optimizing therapeutic windows [138]. Integrating inhibitors with microbiota-ferroptosis axis strategies (e.g., balancing SCFA-driven pro-ferroptotic effects) could mitigate collateral damage and enhance combination immunotherapy efficacy in CRC, aligning with personalized medicine approaches [38,139].

### 4.4. Combination Strategies with Immune Checkpoint Blockade

Combining ferroptosis induction with anti-PD-1/PD-L1 therapy seeks to exploit complementary biology: ferroptosis can increase antigen release and DAMPs, while ICIs sustain CTL function that further downregulates tumor SLC7A11 via IFN-γ. Optimal sequencing is likely crucial: in many models, a priming phase that remodels the microbiota to favor SCFA producers (or direct microbial depletion of IDA producers) followed by carefully dosed tumor-targeted ferroptosis induction and then checkpoint blockade yields the best immune-sensitizing effect [94,140]. Overly aggressive systemic ferroptosis induction before establishing effector T-cell resilience risks collateral immune impairment. Sequencing optimization should consider pharmacodynamic monitoring: microbiome modulation requires 2–3 weeks for ecological stabilization, followed by ferroptosis inducer priming (5–7 days) before ICI initiation [141].

Temporal separation means triggering ferroptosis at a moment when effector T-cell counts are high and their oxidative-stress defenses have been reinforced, for example through IL-2/IL-15 cytokine support or selective antioxidant rescue aimed at T cells [142].

Selective immune-cell protection can be achieved by exploiting metabolic differences—such as unequal reliance on NADPH pools—between tumor and immune cells to create rescue agents that shield T cells while leaving tumor cells unprotected. These ideas are still experimental yet are pivotal for clinical translation [143,144]. New mitochondria-targeted antioxidants (e.g., MitoQ) selectively protect T cells from lipid peroxidation without compromising tumor ferroptosis [145]. Ferroptosis-resistant CAR-T cells engineered with GPX4 overexpression show enhanced persistence in ferroptotic microenvironments [146].

### 4.5. Tumor-Targeted Delivery Platforms

Nanoparticle and biomaterial platforms enable colocalization of ferroptosis inducers, iron modulators, and immunomodulatory agents within the tumor microenvironment or colonic lumen—minimizing systemic exposure and preserving immune effectors [147]. Examples include tumor-homing liposomes that co-deliver GPX4 inhibitors plus PD-L1 antibodies, iron oxide nanoparticles that catalyze Fenton reactions locally, and hydrogel matrices for rectal/topical delivery in localized disease. Coupling these platforms with microbiota modulation (e.g., probiotic payloads or enzymes that degrade IDA) offers attractive multi-modal therapeutic payloads [148,149]. Microbiome-responsive systems are emerging, including probiotic-coated nanoparticles that release payloads in response to microbial enzymes (e.g., azoreductase-triggered release in the colon) [150]. Oral hydrogel microcapsules can protect probiotics from gastric acid and target release to specific gut regions.

### 4.6. Biomarker-Driven Patient Selection and Monitoring

Robust biomarkers will be essential for stratifying patients and monitoring on-treatment biology:

Microbiome signatures include the presence or absence or relative abundance of IDA-producing taxa such as *P. anaerobius*, enrichment of SCFA-producers, and microbial gene pathways for tryptophan metabolism or polyamine biosynthesis [151]. Tumor molecular markers encompass expression of SLC7A11, GPX4, FSP1, ACSL4, NRF2 pathway activation, and intratumoral labile iron content [152,153]. Immune contexture covers baseline CD8^+^ T-cell infiltration, PD-L1 expression, and Treg frequency [154]. Functional assays comprise lipid peroxidation readouts like BODIPY-C11 staining, circulating oxidized lipid species such as 4-HNE adducts, and metabolomic profiles including IDA, SCFAs, and CoQ redox state [155].

Prospective trials should integrate longitudinal stool, blood, and tumor sampling for correlative studies to link interventions with metabolic, immunologic, and clinical outcomes [156,157]. Novel non-invasive biomarkers include ferroptosis-specific PET tracers (e.g., [^18^F]FSP1 inhibitors) and fecal volatile organic compound profiles reflecting microbial metabolic activity [158]. Machine learning algorithms integrating multi-omic biomarkers show promise for predicting patient-specific responses [159].

### 4.7. Safety Considerations and Mitigation of Adverse Effects

Off-target ferroptosis of nonmalignant tissues (intestinal epithelium, cardiomyocytes, immune cells)—mitigate via targeted delivery, dose optimization, and intermittent scheduling [160]. Promotion of immunosuppression through M2 macrophage polarization or enrichment of ferroptosis-resistant Tregs—mitigate by combining with macrophage-reprogramming agents (e.g., CSF1R inhibitors) or Treg-targeting modalities [161]. Microbial translocation and infection risk following aggressive microbiota modulation—mitigate via donor screening (for FMT), phage specificity, and close clinical monitoring [162]. Metabolic compensation and resistance (e.g., NRF2 activation upon oxidized lipid uptake)—mitigate by co-targeting compensatory antioxidant pathways (FSP1, ALDHs) informed by biomarker dynamics [163]. Emerging safety strategies include on-demand ferroptosis induction using optogenetic systems controlled by external light arrays. Microbiome-sparing antibiotics (e.g., ribociclib) show selective antimicrobial activity against pathobionts while preserving commensals [164].

### 4.8. Clinical Translation: Trial Design Considerations

We propose an adaptive, biomarker-enriched trial framework for early clinical evaluation:

Population: pMMR/MSS metastatic CRC patients who are refractory to standard therapies and display a microbiome signature predictive of ferroptosis resistance (e.g., enrichment of IDA producers) or low baseline CD8^+^ infiltration.

Arms: (A) microbiota modulation (defined consortium or FMT) → tumor-targeted ferroptosis inducer → anti-PD-1; (B) control arm with anti-PD-1 ± standard-of-care.

Endpoints: primary safety and objective response rate (ORR); secondary progression-free survival (PFS), overall survival (OS), and translational end points including stool microbiome shifts, tumor lipid peroxidation, and immune infiltration.

Correlatives: serial stool metagenomics/metabolomics, tumor biopsies for SLC7A11/GPX4/FSP1 expression and BODIPY lipid peroxidation assays, and peripheral immune phenotyping. Adaptive rules would allow enrichment for subgroups showing early biological efficacy. Platform trial designs (e.g., BATTLE-3) permit dynamic treatment arm modifications based on interim biomarker analyses. N-of-1 microbiome-guided approaches are being explored for ultra-personalized therapy sequencing [165].

**Figure 3:**
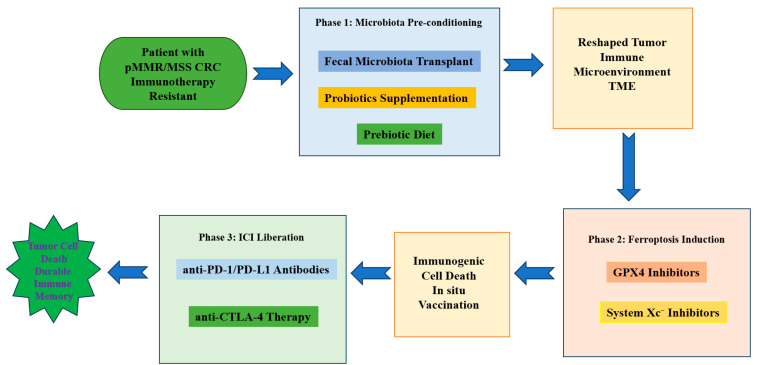
Integrated therapeutic strategies to sensitize pMMR/MSS CRC to immune checkpoint blockade via modulation of the microbiota–ferroptosis axis. A conceptual figure showing—center—tumor cell ferroptosis state; surrounding nodes indicating microbiota modulation (probiotics/FMT/engineered strains), dietary interventions, pharmacologic ferroptosis inducers (system Xc^−^/GPX4/FSP1 inhibitors), tumor-targeted delivery systems (nanoparticles, hydrogels), and checkpoints (anti-PD-1), with arrows indicating synergistic interactions and biomarker readouts. Abbreviations: pMMR, mismatch repair proficient; MSS, microsatellite stable; CRC, colorectal cancer; TME, tumor microenvironment; ICI, immune checkpoint inhibitor; PD-1, programmed cell death protein 1; PD-L1, programmed death-ligand 1; CTLA-4, cytotoxic T-lymphocyte-associated protein 4; GPX4, glutathione peroxidase 4; System Xc^−^, cystine/glutamate antiporter.

A schematic summary of integrated therapeutic approaches targeting the microbiota–ferroptosis axis is provided below (Figure 3).

## 5. Challenges and Future Directions & Conclusions

### 5.1. Major Challenges

#### 5.1.1. Biological Complexity and Interindividual Variability

The microbiota–ferroptosis–immune axis is intrinsically multilayered. Interindividual differences in microbiome composition, host genetics, diet, prior therapies (including antibiotics), and comorbidities produce highly heterogeneous metabolic milieus and immune set points. These sources of variability complicate reproducible translation: an intervention that raises colonic butyrate and sensitizes tumor cells to ferroptosis in one patient may have muted or even opposite effects in another whose microbiome metabolizes substrates differently or whose tumor exhibits alternative antioxidant compensation (e.g., NRF2 activation or FSP1 upregulation). Thus, interpatient heterogeneity will demand precision strategies informed by multi-omic baseline profiling [88,102,166].

#### 5.1.2. Causal Inference Versus Correlation in Human Studies

Human microbiome studies are frequently associative. Demonstrating causality for specific taxa or metabolites in regulating ferroptosis and ICI response requires rigorous mechanistic validation using gnotobiotic/germ-free animal models, defined microbial consortia, and ex vivo human systems (organoids and tumor–immune co-cultures) [167,168]. Without such causal evidence, clinical interventions risk being empiric and inconsistent.

#### 5.1.3. Differential Sensitivity of Immune and Non-Immune Host Cells

Many ferroptosis-inducing approaches are not cell type–selective. Effector lymphocytes and other immune effectors can be vulnerable to lipid peroxidation; systemic ferroptosis induction therefore risks collateral immune suppression that undermines the therapeutic goal. Designing regimens that spare or rescue immune effectors while selectively targeting tumor cells (through delivery platforms, timing, or metabolic rescue for immune cells) remains a central translational hurdle [169].

#### 5.1.4. Safety and Infection Risk with Microbiome Manipulation

Interventions that substantially remodel the gut microbiome—broad-spectrum antibiotics, FMT, or live biotherapeutics—carry risks of pathogen transmission, dysbiosis-associated complications (including Clostridioides difficile infection), and unintended metabolic consequences [170]. For engineered or live microbial therapeutics, concerns include horizontal gene transfer, uncontrolled colonization, and long-term ecological effects that must be addressed through stringent donor selection, containment strategies, and regulatory oversight.

#### 5.1.5. Adaptive Resistance and Metabolic Compensation

Tumors can evolve compensatory antioxidant pathways (e.g., upregulation of NRF2 targets, enhanced FSP1 activity, or ALDH-mediated detoxification) in response to ferroptotic stress or microbiota-directed therapies [125]. Predicting and preempting such adaptive responses will necessitate combinatorial targeting of parallel antioxidant nodes and dynamic biomarker monitoring.

#### 5.1.6. Biomarker Standardization and Assay Limitations

Reliable, reproducible biomarkers are currently lacking. Assays for intratumoral labile iron, functional lipid peroxidation (e.g., validated BODIPY-C11 protocols), quantification of microbially derived metabolites (IDA, SCFAs) in relevant compartments, and standardized microbiome functional readouts must be developed and harmonized across centers to support patient selection and mechanistic readouts in trials [171].

#### 5.1.7. Regulatory, Manufacturing and Ethical Considerations

Live biotherapeutic products (LBPs), bacteriophages, and engineered commensals fall under complex regulatory frameworks that differ by jurisdiction. Scalable, GMP-grade manufacturing, stability, and quality control for microbial therapeutics pose technical and cost challenges. Ethical issues—microbiome privacy, informed consent for donor-derived products, and long-term monitoring obligations—also require proactive frameworks [172,173].

### 5.2. Recommended Future Directions

#### 5.2.1. Deep Mechanistic Dissection in Tractable Experimental Systems

Prioritize causal studies using germ-free and gnotobiotic mouse models colonized with defined consortia, paired with patient-derived organoids and tumor–immune co-culture systems. Use CRISPR screens (tumor and immune cells) to identify genetic modifiers of ferroptosis sensitivity that interact with microbial metabolites [174]. Employ isotope tracing and redox metabolomics to map metabolic fluxes that feed antioxidant systems (GSH, CoQ, NAD(P)H pools). To address interindividual variability, these models should be diversified to reflect human demographic and genetic diversity (e.g., using humanized mice or multi-ethnic organoid libraries), enabling the identification of patient-specific factors influencing ferroptosis susceptibility [175].

#### 5.2.2. Multi-Omics, Spatial and Single-Cell Profiling in Human Cohorts

Integrate longitudinal stool metagenomics/metatranscriptomics, plasma and fecal metabolomics, tumor bulk and single-cell transcriptomics, and spatial proteomics/metabolite imaging to define mechanistic signatures that predict ferroptosis susceptibility and ICI responsiveness [176]. Spatial approaches will be critical to link microbe-driven metabolites with localized tumor and immune states. Furthermore, advanced machine learning algorithms should be applied to multi-omics data to decipher interindividual differences and develop predictive models for personalized therapy selection, such as stratifying patients based on microbial composition, host genetics, and metabolic phenotypes [177].

#### 5.2.3. Rational Design of Combination Regimens and Delivery Modalities

Advance tumor-targeted delivery platforms (nanoparticles, hydrogels, rectal/topical systems) that colocalize ferroptosis inducers with immunotherapies and microbiota-modulating agents to maximize on-target effects and minimize systemic toxicity [178]. Develop selective immune-protective strategies (cytokine support, transient antioxidant rescue for T cells) to preserve effector function during ferroptosis induction [139]. Personalized approaches should be explored, including the customization of delivery systems based on individual gut microbiome profiles (e.g., using microbiome-responsive nanomaterials) and the tailoring of combination therapies to patient-specific ferroptosis thresholds and immune contexts [179].

#### 5.2.4. Precision Microbiome Engineering and Targeted Depletion

Invest in phage libraries, CRISPR-based antimicrobials, and engineered commensals that can selectively deplete IDA-producing or AHR-activating species while restoring SCFA producers [180]. Preclinical evaluation should include ecological network analyses to ensure durable engraftment and to prevent compensatory blooms of off-target taxa. To overcome interindividual variability, these strategies must be adaptable to individual microbiome structures; for instance, developing modular microbial consortia that can be tailored to restore beneficial taxa based on pre-treatment microbiome screening, and designing phage cocktails that target pathogenic strains specific to a patient’s microbial ecology [181].

#### 5.2.5. Biomarker Development and Adaptive Clinical Trial Platforms

Standardize assays for lipid peroxidation, intratumoral iron, and microbial metabolites. Design adaptive, biomarker-enriched clinical trials (umbrella or platform designs) that permit rapid iteration: window-of-opportunity studies to test biological effects (e.g., changes in tumor lipid peroxidation and T-cell infiltration), followed by expansion cohorts selected on early pharmacodynamic responses [182]. Critically, incorporate personalized biomarker frameworks that account for interindividual variability—such as dynamic monitoring of microbial metabolites, host immune signatures, and ferroptosis-related markers (e.g., GPX4 activity, lipid peroxide levels)—to guide real-time therapy adjustment and enable precision enrollment in trials based on individual risk profiles [23].

#### 5.2.6. Cross-Disciplinary Consortia and Data Sharing

**Figure 4:**
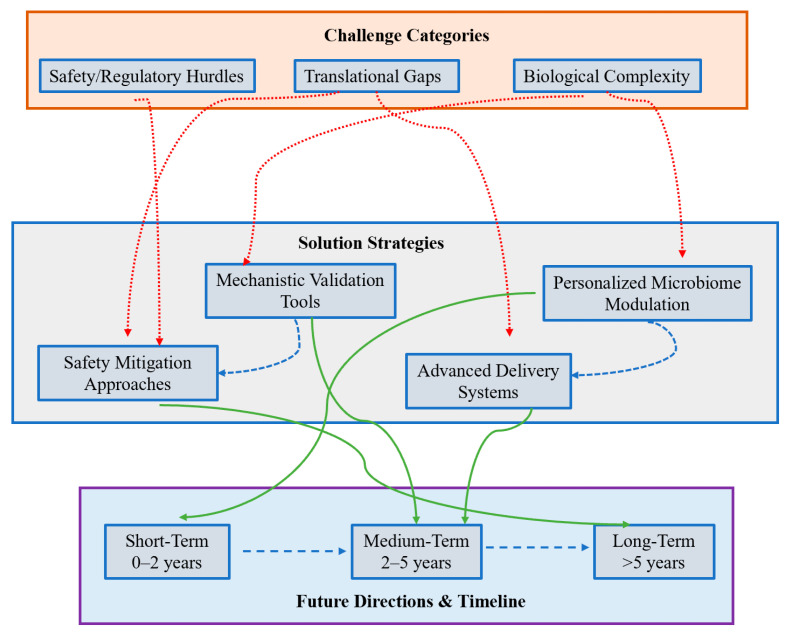
Integrated Roadmap for Targeting the Microbiota-Ferroptosis Axis in CRC Immunotherapy. This schematic outlines a strategic framework to overcome key challenges in leveraging the microbiota-ferroptosis axis. The top tier defines major Challenge Categories (Biological Complexity, Translational Gaps, Safety/Regulatory Hurdles). The middle tier presents corresponding Solution Strategies (e.g., Personalized Microbiome Modulation, Advanced Delivery Systems). The bottom tier details the implementation timeline for Future Directions. Arrow colors indicate functional relationships: Red dashed arrows link challenges to solutions; Green solid arrows connect solutions to timeline milestones; Blue dashed arrows indicate synergistic interactions across the framework.

Assemble multidisciplinary teams (microbiologists, immunologists, tumor biologists, bioengineers, computational biologists, clinicians, and regulatory experts) and create shared repositories for microbiome, metabolomic, and clinical data to accelerate reproducibility and translation [183]. These efforts should prioritize the collection of diverse, large-scale datasets that capture interindividual variability (e.g., across ethnicities, diets, and comorbidities), fostering the development of AI-driven tools for personalized treatment recommendations and regulatory guidelines that accommodate patient-specific therapeutic approaches [184]. This integrated framework is visually summarized in Figure 4, which maps the key challenges, solution strategies, and timeline perspectives discussed above. The roadmap provides a comprehensive visualization of how cross-disciplinary collaboration connects to personalized therapeutic development across short-, medium-, and long-term horizons.

### 5.3. Conclusions

The intersection of gut microbial ecology, ferroptotic cell death, and antitumor immunity reveals a rich and actionable biological landscape with the potential to address one of oncology’s pressing unmet needs—the refractoriness of pMMR/MSS colorectal cancer to immune checkpoint blockade. This review provides the first systematic synthesis bridging these three fields, establishing a novel conceptual framework in which microbial metabolites and community structure dynamically shape tumor redox balance, iron availability, and immune contexture, thereby modulating ferroptosis susceptibility and the immunogenic consequences of tumor cell death. Mechanistic studies to date support this model, highlighting the microbiota-ferroptosis axis as a master regulator of immunotherapy responses.

Translating these insights into durable clinical benefit will require overcoming substantial challenges: demonstrating causality in human-relevant systems, achieving cell type–selective targeting to preserve immune effectors, managing safety risks of microbiome manipulation, and developing robust, standardized biomarkers to guide precision interventions. Critically, while the therapeutic conceptual space is promising, the field must address key gaps: (1) the causal versus correlative nature of microbiome-ferroptosis interactions in human tumors; (2) the paradoxical roles of ferroptosis in simultaneously promoting immunogenic cell death and immunosuppressive remodeling; and (3) the interindividual variability in microbiome composition and host factors that may limit broad applicability. Nonetheless, by combining precision microbiome editing (or restoration), carefully targeted ferroptosis induction, and rational immunotherapy sequencing, it may be possible to convert immunologically “cold” CRCs into responsive, immune-mediated remissions.

As a major critical conclusion, this review emphasizes that targeting the microbiota-ferroptosis axis is not merely an additive strategy but a paradigm shift in overcoming immunotherapy resistance. However, its success hinges on moving beyond generic approaches to personalized intervention frameworks that account for individual microbiome signatures, tumor metabolic states, and immune profiles. Ultimately, progress will depend on rigorous preclinical validation, well-designed clinical studies, and adaptive trial frameworks. If scientific, technical, and regulatory hurdles can be overcome, targeting the microbiota-ferroptosis axis could redefine therapeutic landscapes to broaden the impact of cancer immunotherapy for colorectal cancer patients, though this vision demands coordinated efforts across disciplines to translate mechanistic insights into clinical reality.

## Data Availability

No new data were created or analyzed in this study. Data sharing is not applicable to this article.
